# Compartmentalization and Aggregation of Biomolecular Condensates in Crowded Hydrogels for Enhanced Nucleic Acid Diagnosis

**DOI:** 10.1002/advs.202513938

**Published:** 2025-11-05

**Authors:** Fangbin Xiao, Tao Yang, Mei Fang, Xingyu Lin

**Affiliations:** ^1^ College of Biosystems Engineering and Food Science Zhejiang University Hangzhou 310058 China; ^2^ Binjiang Institute of Zhejiang University Hangzhou 310053 China

**Keywords:** biomolecular condensates, compartmentalization, crowding, hydrogel, molecular diagnosis

## Abstract

Membrane‐less compartments, such as biomolecular condensates, play a crucial role in cellular organization by enhancing enzymatic reaction efficiency through compartmentalization and crowding. This has considerable potential for improving in vitro diagnostics. However, the practical biomedical applications of intracellular microenvironments for in vitro diagnostics remain in their infancy. Herein, inspired by membrane‐less compartments from biomolecular condensates, a crowded hydrogel‐based approach to mimic the intracellular microenvironment through the biocompatible porous structure of hydrogels, thereby promoting the compartmentalization and aggregation of enzymatic reactions to achieve highly sensitive in vitro nucleic acid diagnosis, is proposed. This hydrogel‐based system demonstrates significantly enhanced polymerase chain reaction amplification efficiency through enhanced primer‐template binding and enzyme activity‐mediated single‐stranded DNA extension. This enhancement primarily arises from improved molecular interactions driven by excluded‐volume effects in crowded environments as well as hydrogel compartmentalization. With high enzymatic efficiency, a two‐orders‐of‐magnitude lower detection limit for pathogens is achieved. These findings suggest that crowded hydrogels have the potential to bridge the gap between the intracellular environment and in vitro applications, offering a novel strategy for advanced molecular diagnostics.

## Graphical abstract

A cell‐mimetic hydrogel compartment is proposed to enhance the efficiency of enzymatic reactions. The naturally porous structure of a crowded hydrogel can simulate the intracellular microenvironment, promoting the aggregation of biomolecules and activity of DNA polymerase through excluded volume effects and compartmentalization. This strategy significantly improves the sensitivity of nucleic acid diagnosis.

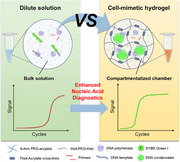

## 1 Introduction

In nature, cells are crowded systems that regulate specific physiological functions by compartmentalizing proteins, RNA, and other biomolecules into distinct cellular compartments.^[^ ^1^ ^]^ This crowded cellular environment is primarily composed of numerous biomolecular condensates. Biomolecular condensates spontaneously form by liquid‐liquid phase separation and function as membrane‐less cellular compartments that support physical partitioning and substrate exchange, and provide a crowded environment for enhanced biochemical reactions. Advanced research has further clarified the processes of biomolecular condensate compartmentalization and their roles in regulating cellular functions.^[^ ^2^, ^3^, ^4^ ^]^ However, studies on synthetic systems and their in vitro biomedical applications remain in the early stages.^[^ ^5^ ^]^

Crowding and compartmentalization are the key regulators of diverse biochemical reactions in densely packed cellular environments. As membrane‐less compartments, biomolecular condensates create micro‐reaction chambers with a crowded environment, which enhances specific cellular functions by isolating and concentrating the reaction components. Furthermore, these compartments enable multiple biochemical reactions to proceed simultaneously and efficiently without cross‐interference. Compartments play crucial roles in regulating DNA replication,^[^ ^6^ ^]^ transcription,^[^ ^7^, ^8^ ^]^ biosynthesis,^[^ ^9^ ^]^ and other cellular processes by promoting the separation and exchange of enzymes, nucleic acids, and other small molecules. Enzymes exhibit different catalytic behaviors in crowded biomolecular condensates compared with that in buffer solutions. Strulson et al. found that the concentration of RNA enriched in crowded condensates increased 3000‐fold, and ribozyme cleavage rate increased 70‐fold.^[^ ^10^ ^]^ Poudyal et al. discovered that membrane‐less compartments significantly increase the activity of various nucleases and reduce their interference with environmental Mg^2+^ concentrations.^[^ ^11^ ^]^ These biomolecular condensate properties offer new strategies for improving enzyme‐mediated nucleic acid diagnostics.

Nucleic acid testing is an important tool in biomedical research and clinical diagnostics.^[^ ^12^, ^13^, ^14^ ^]^ In vitro nucleic acid diagnostics typically rely on enzyme‐mediated nucleic acid amplification and cleavage reactions, such as the polymerase chain reaction (PCR) mediated by DNA polymerase^[^ ^15^, ^16^ ^]^ and nucleic acid cleavage reactions mediated by CRISPR/Cas nucleases.^[^ ^17^, ^18^ ^]^ Traditional enzyme‐mediated in vitro nucleic acid diagnostics are performed in dilute solutions lacking the crowded and compartmentalized environment, which leads to slower enzyme reaction kinetics, susceptibility to inhibitors, and insufficient detection sensitivity. This limits accurate diagnosis and early warning of diseases and pathogen infections.^[^ ^19^ ^]^ In contrast, these natural enzyme‐mediated reactions perform well inside the cells in crowded and compartmentalized environments. Achieving high enzymatic reaction efficiency in an in vitro environment remains a major challenge. Hydrogels have been used to expand PCR‐based nucleic acid diagnostic applications. However, the use of hydrogels to enhance amplification efficiency and detection sensitivity has not yet been reported.^[^ ^20^, ^21^, ^22^ ^]^

Inspired by intracellular membrane‐less compartments (biomolecular condensates), and the water‐rich, 3D porosity of hydrogels, we propose using a crowded hydrogel to mimic the intracellular microenvironment, thereby promoting biomolecular compartmentalization and aggregation to enhance in vitro nucleic acid diagnostics. Compared with biomolecular condensates, which are dynamically regulated in physiological contexts, hydrogels offer a more robust, controllable, and engineered in vitro system for emulating the efficient enzymatic reaction environment found intracellularly. Moreover, similar to biomolecular condensates in cells, the 3D nanoporous structure enables redistribution and compartmentalized aggregation of biomacromolecules, thereby significantly improving DNA polymerase‐mediated PCR amplification efficiency. This biomimetic hydrogel approach has broad potential for nucleic acid analysis, particularly for the efficient detection of low‐abundance targets in complex samples, including the research fields of pathogen screening, disease diagnosis, and gene sequencing.

## 2 Results and Discussion

### 2.1 The crowded Hydrogel Enhanced Nucleic Acid Diagnostics

**Figure 1:**
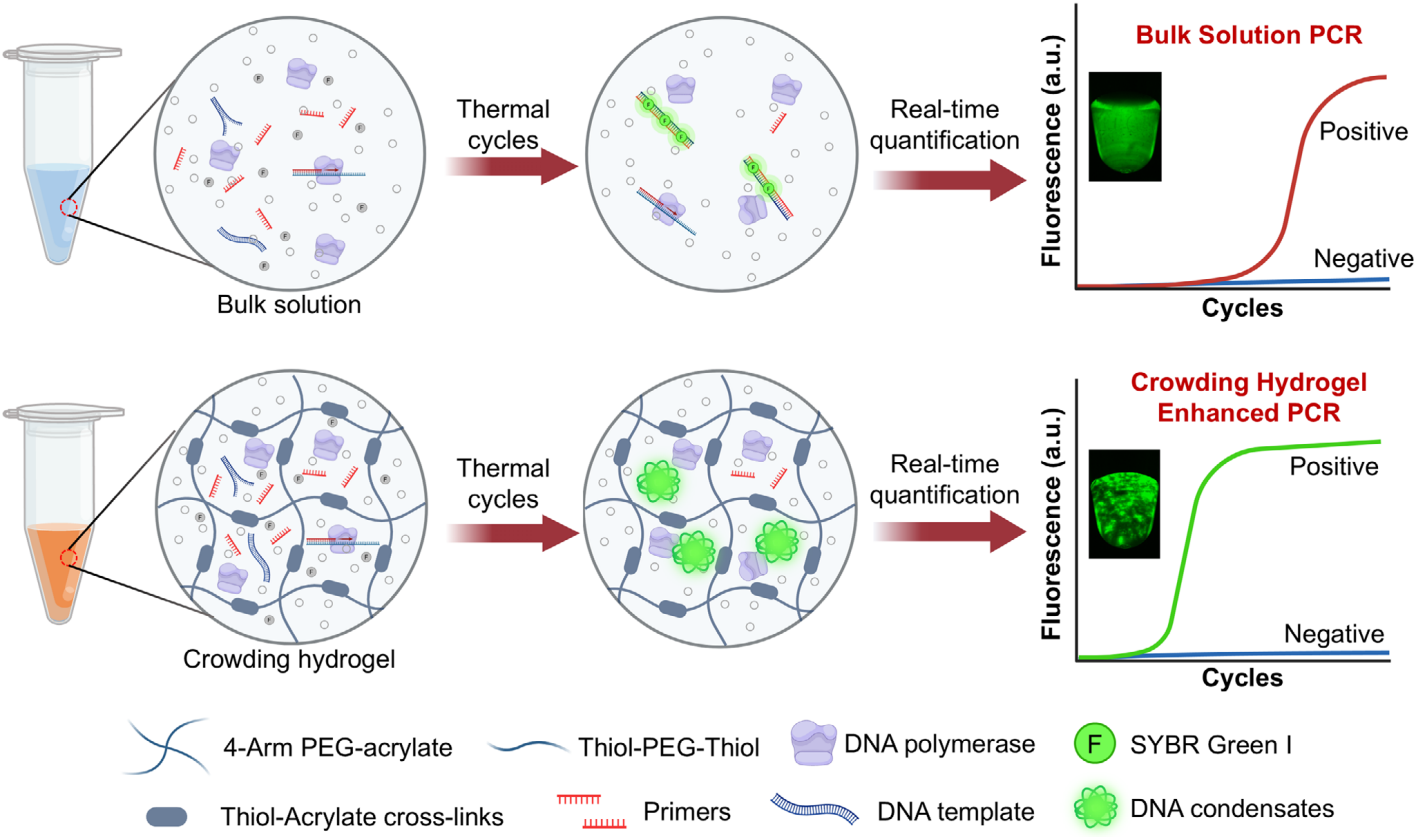
Schematic illustration of the crowded hydrogel enhanced nucleic acid diagnostics. The inset shows end‐point fluorescence images of the solution and hydrogel reaction system in the PCR tube. Created in BioRender.com.

The hydrogel accelerated the enzymatic reaction kinetics, and promoted high amplification efficiency and detection sensitivity (**Figure** **1**). A mixture containing PCR reagents, a DNA template, and two types of hydrogel monomers were added to a PCR tube, and the hydrogel network was automatically crosslinked within 2 min via the Michael addition reaction between the 4 arm polyethylene glycol acrylate (4‐Arm PEG‐AC) and thiol‐polyethylene glycol‐thiol (SH‐PEG‐SH) under alkaline conditions.^[^ ^23^ ^]^ After cross‐linking, the hydrogel formed a natural 3D porous compartmentalized structure that resembled intracellular compartments, and PCR components automatically aggregated in each nanopore. In the conventional assay, PCR components were distributed in the bulk solution. During thermal cycling, locally concentrated PCR components fully contacted and reacted in the compartmentalized nanoreactors of the hydrogel, and PCR amplification was monitored in real time by pre‐adding SYBR Green I dye to the mixture. The PCR amplification efficiency in the crowded hydrogel was significantly improved compared with that of the bulk solution, which was due to the fact that primer‐template binding and DNA polymerase activity were enhanced in the crowded environment. The PCR on hydrogels detected lower concentrations of DNA targets. Therefore, it has substantial potential for high‐sensitivity nucleic acid diagnostics, gene sequencing, and other nucleic acid applications.

### 2.2 Characterization of Crowded Hydrogel

**Figure 2:**
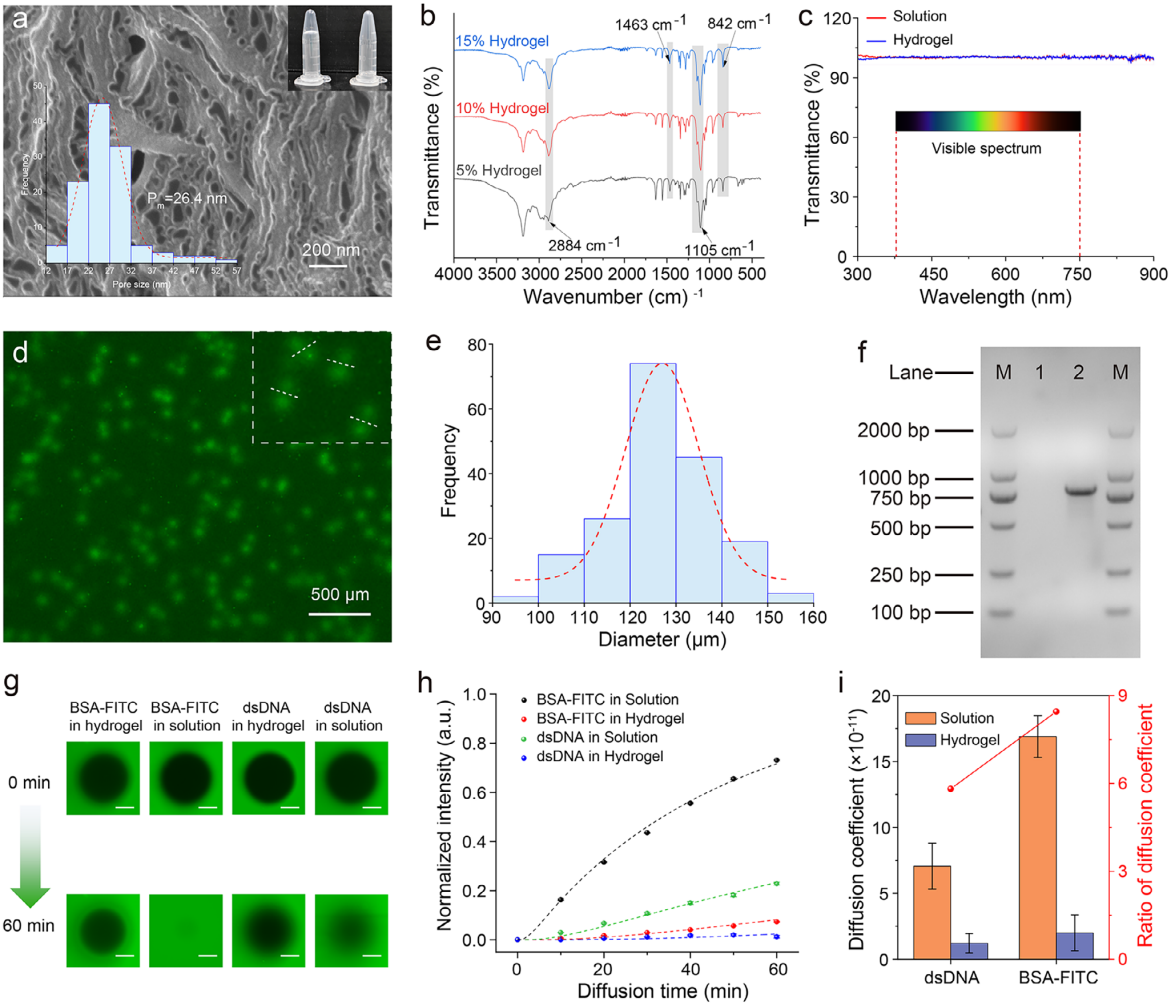
Characterization of hydrogel and biomolecular condensate. a) SEM image and pore size distribution histogram of crowded nanoporous hydrogel. The insert image shows hydrogel (left) and solution (right) reaction systems in PCR tubes. b) ATR FT‐IR spectrum of hydrogel with different concentrations; the gray areas represent the positions of characteristic peaks. c) Transmission spectra of hydrogel and solution. d) DNA condensates formed in hydrogel after PCR amplification with SYBR Green I dye, the insert image is an enlarged view showing that each DNA condensate has clear boundaries. e) Histogram of DNA condensate diameter distribution. f) Agarose gel electrophoresis of DNA condensate. Lane M, 2,000 bp DNA Marker. Lanes 1 and 2 are extracted from the background and fluorescent spots in hydrogel. g) Fluorescence recovery images of BSA‐FITC and dsDNA in hydrogel and solution after photobleaching for 0 and 60 min. Scale bar, 500 µm. h) Fluorescence recovery quantification and diffusion kinetics fitting curves of BSA‐FITC and dsDNA in hydrogel and solution (Bars represent the mean ± SD, *n* = 3). i) Diffusion coefficients of BSA‐FITC and dsDNA in hydrogel and solution (Bars represent the mean ± SD, *n* = 3). ATR FT‐IR, attenuated total reflectance‐Fourier transform infrared; BSA‐FITC, bovine serum albumin labeled with fluorescein isothiocyanate; dsDNA, double‐stranded DNA.

First, the crowded hydrogel was characterized. The PCR mixture containing the two PEG monomers was polymerized in a PCR tube after standing at room temperature for 2 min, and then formed a transparent gel adhering to the bottom of the tube, whereas the aqueous solution dropped down to the tube cap owing to gravity (**Figure** **2**a). The pore size of the hydrogel network is a critical factor that influences the compartmentalization of PCR components. As shown in Figure 2a, the scanning electron microscopy (SEM) image reveals that the pore size of the 10% hydrogel is ≈26.4 nm,^[^ ^24^ ^]^ allowing the PCR components to concentrate in the nanopore compartments. By decreasing or increasing the concentration of hydrogel, the pore size of the hydrogel compartments could be easily adjusted to meet different requirements (Figure S1, Supporting Information). Attenuated total reflectance‐Fourier transform infrared spectrometry (ATR FT‐IR) showed the functional groups of the hydrogels at different concentrations (Figure 2b). The characteristic absorption peaks at 842 and 2884 cm^−1^ represented the stretching vibrations of the ─CH_2_ group in PEG,^[^ ^25^ ^]^ 1105 cm^−1^ represented the stretching mode of the C─S bond formed by the reaction between the thiol and acrylate groups, and 1463 cm^−1^ represented the stretching vibration of the carboxylate group associated with PEG. Moreover, the hydrogel demonstrated almost 100% transmittance within the wavelength range of 300–900 nm, indicating that the hydrogel network does not interfere with optical detection (Figure 2c).

Following PCR amplification in the crowded hydrogel, clearly defined fluorescent spots were observed (Figure 2d). These spots had uniform circular shapes and diameters of ≈126.6 µm (Figure 2e). Fluorescent spots were extracted by gel punching, followed by agarose gel electrophoresis. As shown in Figure 2f, these fluorescent spots were DNA condensates formed by the aggregation of PCR amplification products within the membrane‐less compartments of the hydrogel. To confirm the recruitment behavior, a FAM (Carboxyfluorescein)‐labeled primer was used. As shown in Figure S2 (Supporting Information), these primers tended to be concentrated in the hydrogel, demonstrating the recruitment and enhancement of DNA. Photobleaching and fluorescence recovery experiments were conducted to verify the compartmentalization effect of the nanoporous hydrogel structure. We used bovine serum albumin labeled with fluorescein isothiocyanate (BSA‐FITC; with a molecular weight of ≈68 kDa, similar to that of DNA polymerase) and purified 800 bp of double‐stranded DNA (dsDNA) to observe the nano‐confined diffusion effect in the hydrogel. As shown in Figure 2g,h, the fluorescence recovery rates of BSA‐FITC and dsDNA in the hydrogel were significantly lower than those in solution. The measured diffusion rates of BSA and dsDNA in hydrogels were 8.46 and 5.81‐fold slower, respectively, than those in solution (Figure 2i). Therefore, the diffusion of proteins and DNA was significantly restricted in a crowded hydrogel environment, similar to that in natural cells. The natural compartments formed by the hydrogel network structure segregate the PCR components into nanocompartments, ultimately leading to the formation of individual DNA condensates within these compartments.

### 2.3 Hydrogel‐Enhanced Quantitative PCR (qPCR)

**Figure 3:**
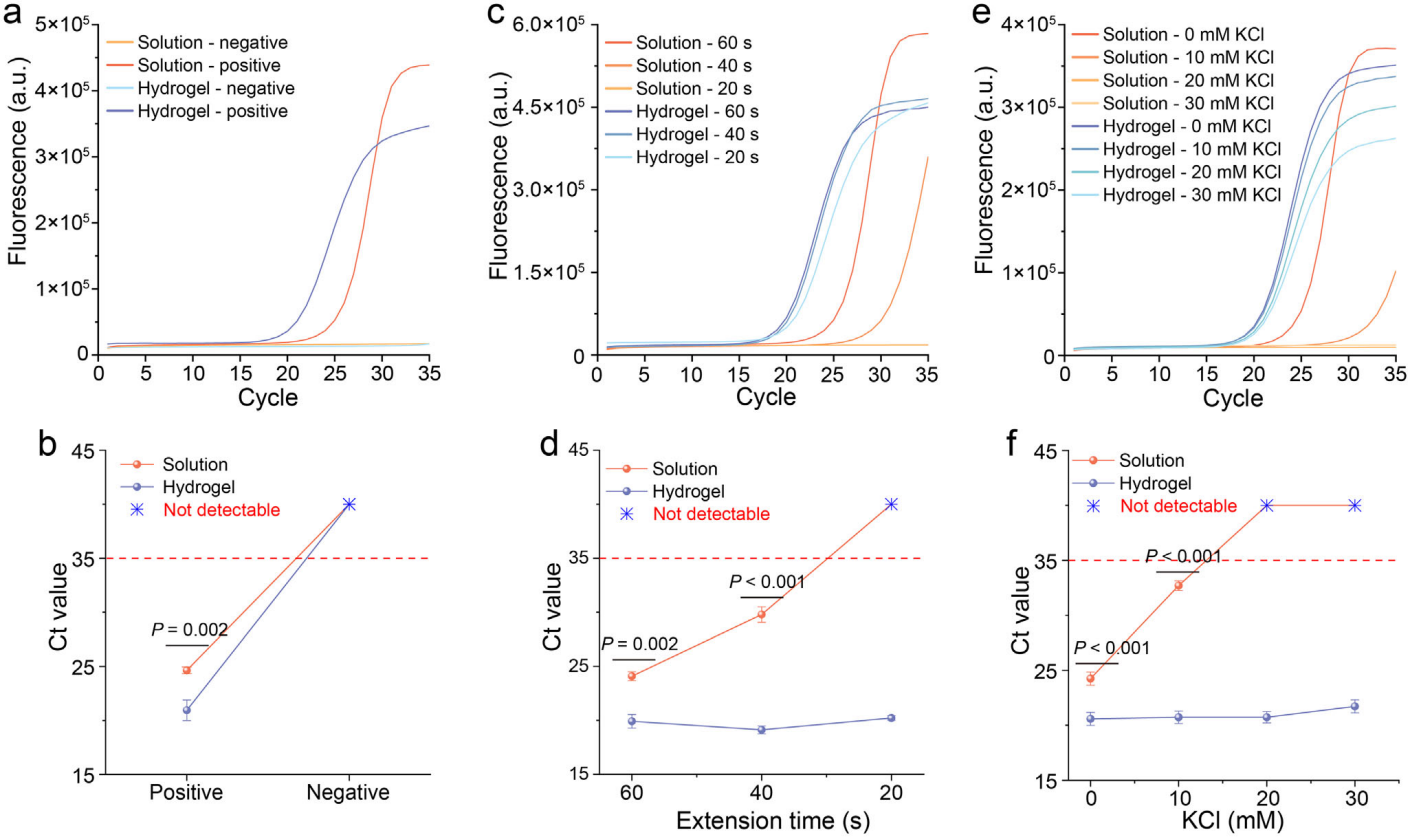
Hydrogel‐enhanced qPCR amplification. Ct values exceeding 35 were defined as undetectable and are indicated by blue asterisks (∗). a,b) Real‐time fluorescence quantification curves and Ct values in the solution and hydrogel (Bars represent the mean ± SD, *n*  =  3). c,d) Effect of extension time on real‐time fluorescence quantitative curve and Ct value (Bars represent the mean ± SD, *n*  =  3). e,f) Effect of KCl on real‐time fluorescence quantitative curve and Ct value (Bars represent the mean ± SD, *n*  =  3). b, d, f were determined using a two‐tailed Student's *t*‐test (*p* < 0.05 was considered statistically significant). Ct, cycle threshold value.

Real‐time qPCR is the transition from qualitative to quantitative analysis. Real‐time qPCR curves of a 112 bp DNA template in aqueous solution and hydrogel are shown in **Figure** **3**a. At the same template concentration, the cycle threshold (Ct value; defined as the cycle threshold time when fluorescence begins to increase) of qPCR in the hydrogel was significantly earlier by 4.34 cycles compared with that in the bulk solution (Figure 3b), indicating that the detection sensitivity of qPCR in the hydrogel was ≈20‐fold higher than that in the bulk solution system. Notably, as the annealing/extension time of the PCR program was gradually shortened, the Ct values in the solution were significantly delayed and could not be detected at 20 s (Figure 3c). In contrast, the Ct values of the hydrogel systems remained almost unchanged (Figure 3d). This suggests that the PCR amplification in the crowded hydrogel compartments was ultrafast and could complete the annealing/extension steps in a shorter time, significantly improving the PCR amplification efficiency.

KCl is a common inhibitor of PCR amplification.^[^ ^26^ ^]^ These inhibitory effects are readily tolerated by natural cell organisms but cause large decreases in enzyme activity when assayed in vitro. Record et al. reported that under high‐osmolality conditions, the total intracellular potassium concentration of *E. coli* can reach up to 0.9 m, yet cellular replication and growth rates remain relatively unaffected.^[^ ^27^ ^]^ Therefore, we tested the PCR amplification under varying KCl concentrations in both aqueous solutions and cell‐mimicking hydrogel environments. As shown in Figure 3e, the amplification curve in the aqueous solution was significantly delayed as the KCl concentration increased, with complete inhibition observed at 20 mm KCl. In contrast, PCR amplification of the hydrogel successfully amplified the template regardless of KCl presence, with the Ct value remaining nearly unchanged (Figure 3f). Therefore, high concentrations of KCl inhibit the annealing/extension steps in PCR, and crowded hydrogel compartments mitigate this inhibition, similar to that in naturally crowded cellular environments. This may be because the crowded internal environment of the hydrogel alleviates the inhibitory effect of K^+^ on DNA polymerase activity, similar to natural cells that induce macromolecular crowding to maintain normal gene expression at high intracellular K^+^ concentrations.^[^ ^28^ ^]^ These findings indicate that nucleic acid amplification in hydrogel compartments is robust and efficient, maintaining high amplification efficiency and gene quantification capabilities, even in complex environments.

### 2.4 Amplification of Template with Different Lengths in Hydrogel

**Figure 4:**
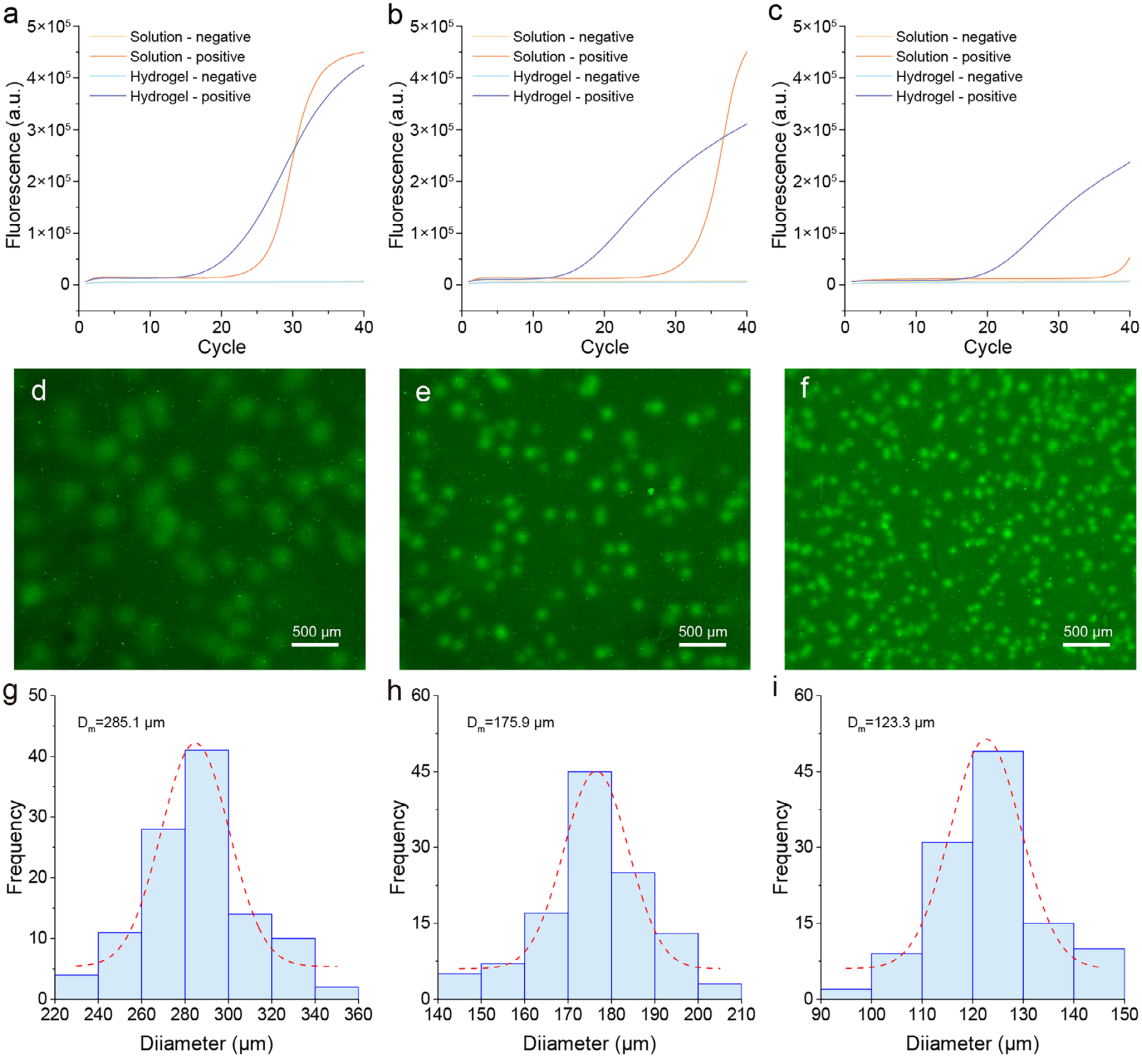
Amplification of dsDNA with different lengths in hydrogel. a–c) Real‐time fluorescence quantification curves of DNA templates with lengths of 423 (a), 605 (b), and 800 bp (c) in solution and hydrogel. d–f) Fluorescence images of DNA condensates amplified using dsDNA templates with lengths of 423 (d), 605 (e), and 800 bp (f). g–i) Diameter distribution histograms of DNA condensates with lengths of 423 bp (g), 605 bp (h), and 800 bp (i). dsDNA, double‐stranded DNA.

In high‐throughput qPCR‐based gene analysis, shorter DNA fragments are typically designed for amplification to ensure high amplification efficiency and specificity. Long‐range amplification products are commonly used for the precise genetic identification of microbial diversity^[^ ^29^ ^]^ and mitochondrial DNA heterogeneity.^[^ ^30^ ^]^ However, longer DNA templates increase the complexity because of the increased difficulty in primer binding and the burden of DNA polymerase extension. As shown in **Figure** **4**a–c, as the template length increased from 423 to 800 bp, the Ct value of the qPCR amplification curve in the aqueous solution was progressively delayed, indicating that the amplification of longer DNA templates was significantly hindered. In contrast, the amplification of longer templates in the hydrogel retained high efficiency (Figure 4a–c). As shown in Figure 4d–i, as the amplicon length increased from 423 to 605 to 800 bp (Figure S3, Supporting Information), the diameter of the DNA condensates gradually decreased, measuring 285.1, 175.9, and 123.3 µm, respectively. The presence of DNA condensate spots generated by compartmentalized in‐gel PCR amplification enables the digital counting of endpoint results for absolute quantification.^[^ ^21^ ^]^ Owing to the compartmentalized structure of the hydrogel, PCR are effectively partitioned into numerous nanoscale reaction nanopores, thereby preventing cross‐interference between individual reactions and ensuring high efficiency and uniform in situ amplification.

### 2.5 Mechanism of Enhanced PCR in Hydrogel

**Figure 5:**
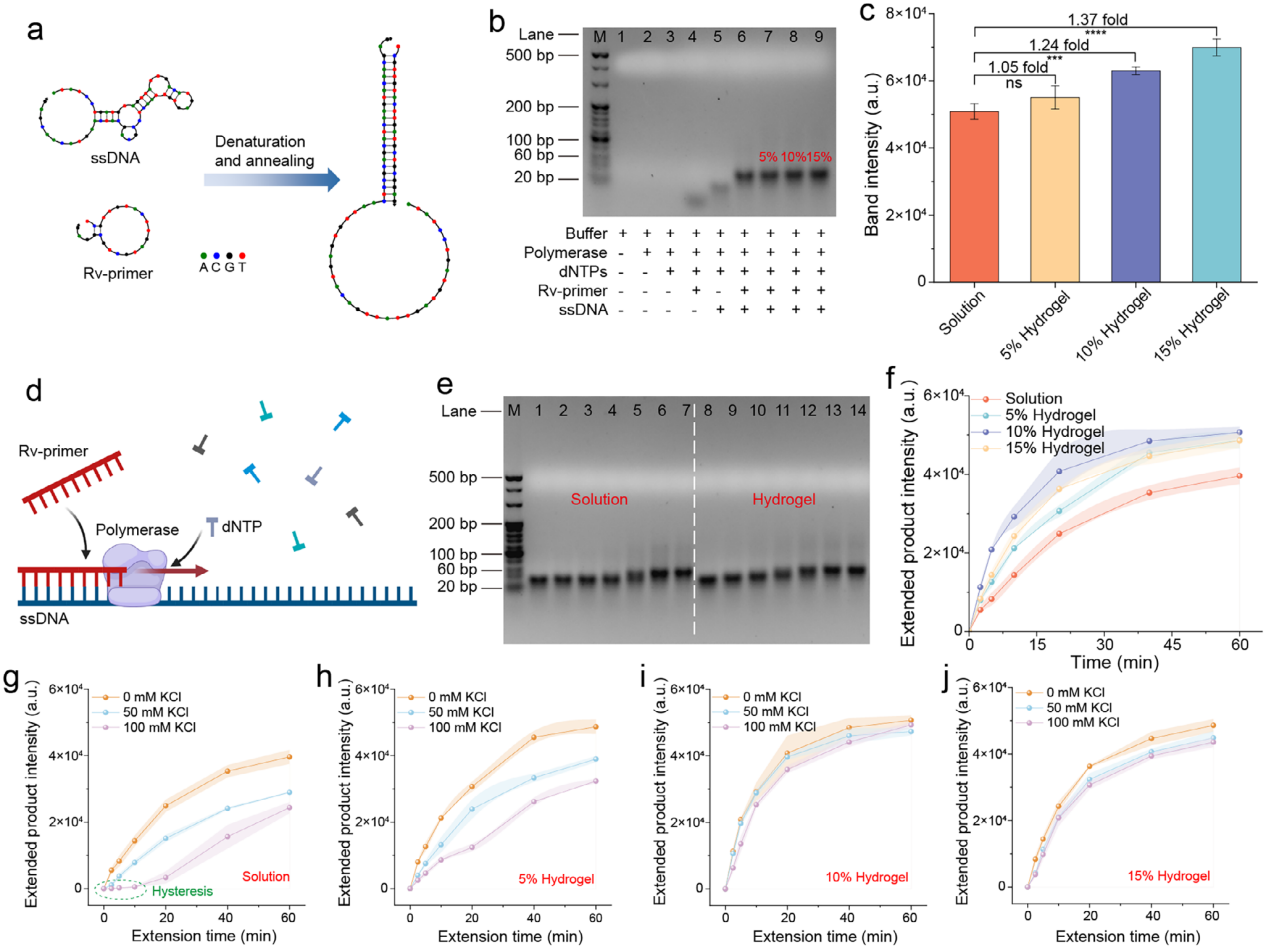
Mechanism of enhanced PCR in hydrogel. a) Schematic diagram of reverse primers binding to template ssDNA. b) Agarose gel electrophoresis image of different components before PCR extension. Lane M, 20 bp DNA ladder. Lanes 1–9 were different components, where lanes 6, 7, 8, and 9 were in solution, 5% hydrogel, 10% hydrogel, and 15% hydrogel systems, respectively. c) Measurement of band intensity of primer‐template binding (Bars represent the mean ± SD, *n*  =  3) and a significant difference was determined using one‐way ANOVA with multiplicity adjusted *P* value (^***^ *p* < 0.001; ^****^ *p* < 0.0001; ns: *p* ≥ 0.05). d) Schematic diagram of polymerase‐mediated ssDNA extension reaction. e) Agarose gel electrophoresis image of extended products at different times. Lane M, 20 bp DNA ladder. Lanes 1–7 and 8–14 represent extension products with extension times of 0, 2.5, 5, 10, 20, 40, and 60 min, respectively. f) Extension kinetics of ssDNA in different systems (color‐filled bars represent the mean ± SD, *n* = 3). g–j) Effects of KCl on ssDNA extension kinetics in different systems (color‐filled bars represent the mean ± SD, *n* = 3).

We investigated the mechanism underlying the enhanced PCR amplification efficiency observed in the crowded hydrogels. PCR amplification involves three phases: denaturation, annealing, and extension. The speed of PCR primarily depends on two factors^[^ ^31^ ^]^: 1) the efficiency of primer‐template binding during annealing and 2) extension rate of the polymerase during the extension step. Therefore, we used a single‐stranded DNA (ssDNA) template and reverse primer (Rv‐primer) to explore primer‐template binding in different environments (**Figure** **5**a). After the mixture was denatured at 95 °C and annealed at 60 °C, the free energy of the Rv‐primer‐ssDNA binding was −22.75 kcal mol^−1^ (Figure S4, Supporting Information), indicating a strong binding force between the Rv‐primers and the ssDNA template. Agarose gel electrophoresis (Figure 5b) revealed distinct bands, indicating successful primer‐template binding. According to the quantitative analysis, the intensity of primer‐ssDNA binding in crowded hydrogels was 1.37‐fold higher than that in the aqueous solution (Figure 5c). Therefore, a crowded hydrogel environment significantly enhanced primer‐template binding. These observations align with previous studies, indicating that biomolecular condensate environments can enhance nucleic acid interactions.^[^ ^32^ ^]^

DNA polymerase extension is another crucial factor affecting PCR amplification efficiency because it is responsible for sequence synthesis during the extension phase. We designed an ssDNA extension experiment using DNA polymerase. As shown in Figure 5d, the Rv‐primers bound to the ssDNA template were extended in the presence of DNA polymerase, and the extension products at different time points were quantified using agarose gel electrophoresis. Within the first 20 min of ssDNA extension, the product bands in the crowded hydrogels were greater than those in the aqueous solution (Figure 5e). Quantitative analysis of the bands revealed that the intensities of the extension products linearly increased before reaching saturation (Figure 5f). The band intensities of the extension products in the hydrogels were significantly higher than those in the aqueous solution. Specifically, the extension rates (defined as the slope of the linear region in the extension kinetics curve) for the 5%, 10%, and 15% hydrogels were 2.66, 4.87, and 3.14 times that of the aqueous solution, respectively. Polymerase activity testing using a commercial kit showed that the nucleotide synthesis speed in the hydrogel was 2.15‐fold that observed in the aqueous solution (Figure S5a, Supporting Information). These results indicate that the compartments of the hydrogel can significantly enhance DNA polymerase extension activity, thereby improving PCR amplification efficiency. The reduction in the extension products at higher hydrogel concentrations may be due to the dense network structure, which limits the diffusion of the reaction components with DNA polymerase. Similar enhancements in enzyme activity were observed for other proteases. Compared with the aqueous solution, the trans‐cleavage activities of Cas12a and Cas13a in the hydrogels were enhanced by 5.34 and 2.33 times, respectively (Figure S6, Supporting Information). Although we did not test a broader range of proteases, these results indicate that crowded hydrogels have a pronounced enhancement in the enzyme activity for some proteases.

KCl significantly inhibited polymerase activity and reduced the yield of PCR products.^[^ ^33^, ^34^ ^]^ When we introduced KCl as an inhibitor in the ssDNA extension (Figure 5g–j), the extension speed in aqueous solution gradually decreased, whereas it remained nearly unchanged in the 10% and 15% hydrogels. At an inhibitor concentration of 50 mm KCl, the extension speed in the 10% hydrogel was 6.08 times that in aqueous solution, demonstrating a robust resistance to KCl inhibition in crowded hydrogels. Notably, at a KCl concentration of 100 mm, the extension in aqueous solution was almost completely inhibited at first with an obvious “hysteresis”, and gradually recovered with time (Figure 5g; Figure S5b, Supporting Information). This hysteresis may be due to the delayed activation of polymerases to adapt to extreme environments. In the hydrogel, the extension still performed well and extension rate in the 10% hydrogel was 37.67 times that in the solution. Therefore, the compartments in the crowded hydrogel provided a cell‐like internal environment, allowing the reactions to proceed independently and stably without substantial cross‐interference.

Enzymes exhibit enhanced activity and stability in crowded and compartmentalized environments. For instance, Bishnu et al. reported that molecular crowding could accelerate the cleavage activity of RNase and reduce its dependence on Mg^2+^ concentration to near‐physiological levels.^[^ ^35^ ^]^ Marcos et al. demonstrated that local environments are key regulators of enhanced enzymatic activity in biomolecular condensates, with a larger enzymatic rate enhancement in nanoscale condensates than that in microscale condensates.^[^ ^36^ ^]^ We propose that in our cell‐like hydrogel system, the crowded microenvironment increases the effective local concentration of reactants through the excluded‐volume effect,^[^ ^37^ ^]^ which increases the possibility of molecular collision frequency and binding, thereby enhancing the combination of primers and templates and the rate of extension (Figure 5c,f). A crowded environment might favor intermolecular association reactions but not dissociation reactions.^[^ ^38^ ^]^ The critical step in most enzymatic reactions requires binding between the enzyme (e.g., polymerase) and substrate (e.g., template), therefore, the PCR reaction would be enhanced in our crowded hydrogel environment. In contrast, hydrogel compartmentalization functions as a nanoreactor with high local concentrations of biomolecules, restricting the diffusion of reaction intermediates and polymerase‐associated macromolecules, thereby enhancing nano‐confined DNA polymerase‐mediated ssDNA extension activity. These viewpoints are supported by confined diffusion experiments, where the diffusion rates of proteins and nucleic acids in the hydrogel decreased by 8.46‐ and 5.81‐fold, respectively (Figure 2g–i). Within the hydrogel compartments, each enzymatic reaction occurred independently and stably within individual nanopores, preventing interference and eliminating competition or inhibition.^[^ ^39^ ^]^ Under high KCl concentration conditions, the elevated salt concentration typically inhibits binding kinetic among the template, primer, and enzyme, leading to the delayed extension curves with an obvious “hysteresis” and requiring more time to recover the extension.^[^ ^40^ ^]^ However, the crowded and compartmentalized hydrogel environment enhances molecular interactions, promoting efficient primer‐template binding and DNA extension despite high‐salt conditions. Consequently, compared with a diluted aqueous environment, the compartmentalized aggregation provided by the hydrogel allows DNA polymerase‐mediated ssDNA extension to tolerate higher concentrations of K⁺ inhibitors (Figure 5g–j). Therefore, the proposed hydrogel system provides a crowded, compartmentalized micro‐reaction environment similar to intracellular conditions, which can significantly enhance the PCR amplification efficiency and offer a promising solution for highly sensitive in vitro nucleic acid diagnostics. To confirm that the enhanced performance was indeed due to cell‐mimicking crowding of the hydrogel environment, control experiments were conducted. As shown in Figure S7a (Supporting Information), under identical conditions (with the same components and hydrogel monomers) but without hydrogel gelation, no crowded hydrogel compartments were formed, and there were no DNA condensate spots. Therefore, we did not observe any PCR enhancement (Figure S7b, Supporting Information), confirming that the PCR enhancement was due to crowded hydrogel compartments after cross‐linking.

### 2.6 Application of the Crowded Hydrogel in Enhanced Nucleic Acid Diagnostics

**Figure 6:**
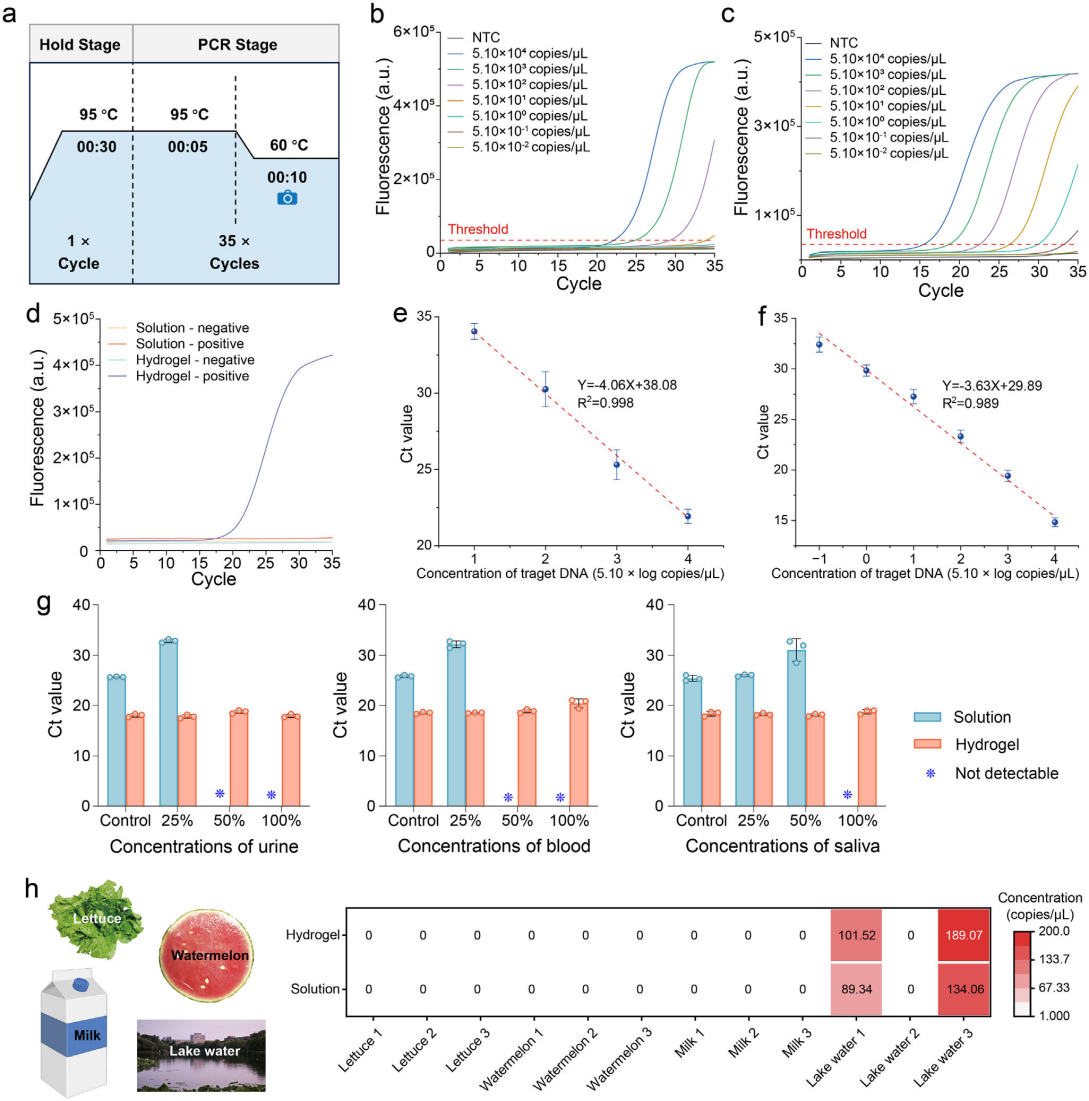
Application of the crowded hydrogel in enhanced nucleic acid diagnostics. a) Temperature and time settings for of the fast real‐time PCR program. b,c) Real‐time fluorescence quantification curves of target DNA at different concentrations in the solution (b) and hydrogel (c). d) Rapid real‐time fluorescence quantification curves in the solution and hydrogel. e,f) Standard curves and sensitivity for detecting *Salmonella* Typhi using qPCR in the solution (e) and hydrogel (f) (Bars represent the mean ± SD, *n* = 3). g) Effects of different concentrations of blood, urine, and saliva clinical samples on qPCR in the solution and hydrogel (Bars represent the mean ± SD, *n* = 3). h) Real sample detection, the heat map showing the DNA concentration in 12 different samples measured using standard qPCR and hydrogel‐enhanced qPCR. Ct, cycle threshold value.

Currently, qPCR detection typically requires more than 1 h, which is insufficient for the urgent need for rapid pathogen screening, such as during the influenza and SARS‐CoV‐2 pandemics. Therefore, we investigated the feasibility of rapid qPCR amplification using crowded hydrogels. We developed a two‐step qPCR program with a minimal extension time, completing the entire amplification process in only 26 min (**Figure** **6**a). As shown in Figure 6d, even when the annealing/extension time was shortened to 10 s, the Ct value of the hydrogel remained almost unchanged (Figure 3a); however, no amplification product was detected in the aqueous solution (Figure S8, Supporting Information). Therefore, the crowded hydrogel compartments can significantly shorten the turnaround time of qPCR and improve detection efficiency. In contrast to previous reports that used the high thermal conductivity and positive charge enrichment effect of nanomaterials for rapid qPCR amplification,^[^ ^41^ ^]^ we proposed a novel mechanism (the crowded hydrogel) to enhance primer‐template binding and DNA polymerase activity for rapid qPCR. This technology is expected to shorten the turnaround time for the nucleic acid diagnosis of pathogens in clinics and laboratories, thereby providing patients with the best opportunities for treatment. Notably, the total detection time of 26 min was limited to our PCR instrument, as the shortest annealing/extension time was 10 s in our PCR machine (i.e., shorter annealing/extension times of < 10 s was not available). Additionally, the heating and cooling speed of the PCR instrument substantially affects the total detection time, which was also included in the 26 min. Therefore, by further reducing the annealing/extension time, and increasing the heating and cooling speeds, the detection time could be further shortened.

The sensitivity and accuracy of nucleic acid diagnosis are critical parameters for evaluating qPCR technology. As a proof‐of‐concept, the common pathogen, *Salmonella* Typhi, which can cause high morbidity and mortality in the public health system, was chosen as the detection target.^[^ ^42^, ^43^ ^]^ Specific primers were designed for the *sii*A gene of *Salmonella* Typhi. Genomic DNA extracted from *Salmonella* Typhi was quantified using a commercial digital PCR, and qPCR was performed in aqueous solution and hydrogel simultaneously. The amplification curves for various concentrations of target DNA displayed a clear gradient (Figure 6b,c). The limit of detection (LOD) for qPCR in aqueous solution was 5.10 × 10^1^ copies/µL, while the crowded hydrogel‐enhanced qPCR achieved an LOD of 5.10 × 10^−1^ copies/µL, representing a two‐order‐of‐magnitude improvement. Linear fitting of the Ct values against the target concentrations yielded standard curves for qPCR in the aqueous solution and hydrogel as Y = −4.06X + 38.08 (R^2^ = 0.998) and Y = −3.63X + 28.89 (R^2^ = 0.989), respectively (Figure 6e,f). These results indicate that the nanoscale compartments of the crowded hydrogel significantly enhanced the sensitivity of qPCR technology, offering a broader detection range. Compared with previous qPCR technologies using conventional PCR instruments (**Table** **1**), the crowded hydrogel‐enhanced qPCR presented in this study demonstrated superior sensitivity and accuracy.

**Table 1:** Comparison of enhanced PCR technologies using a conventional heat block.

Name	Sensitivity	PCR duration	Enhancement mechanism	Refs.
Microfluidic thermal control system stabilized PCR	193 copies/µL	>41 min	Precise temperature control	[44]
MIR^a)^ light‐enhanced PCR	NR^b)^	1 h	Vibrational strong coupling between MIR photons and carbonyl groups	[45]
THz laser‐assisted PCR	NR^b)^	>33 min	Resonant coupling between MIR and DNA carbonyl vibrations	[46]
Magnetofluidic immuno‐PCR	2 ng mL^−1^	30min	Magnetic enrichment	[47]
Core–shell Au nanoparticles enhanced PCR	10 copies	28 min	Electrostatic adsorption and high thermal conductivity	[41]
Crowded hydrogel‐enhanced PCR	0.51 copies/µL	26 min	Compartmentalization and aggregation of biomolecular condensates	This study

^a)^ MIR, middle infrared;

^b)^ NR, not reported.

We subsequently evaluated the repeatability (Figure S9, Supporting Information), stability (Figure S10, Supporting Information), and instrument compatibility (Figure S11, Supporting Information) of hydrogel‐enhanced PCR. The results demonstrated that the established method was applicable to various PCR hardware, exhibited high repeatability, and maintained its detection performance even after storage at 25 °C for up to 72 h. To further validate the practicality of hydrogel‐enhanced PCR in complex matrices, we evaluated its performance using blood, urine, and saliva samples. As shown in Figure S12 (Supporting Information) and Figure 6g, the detection performance of the hydrogel PCR remained consistent across tests involving various concentrations of blood, urine, and saliva, with no significant delay in the Ct values. In contrast, conventional PCR conducted in aqueous solutions exhibits varying degrees of inhibition in these clinical sample matrices. These results indicate that hydrogel‐enhanced PCR possesses broad resistance to matrix interference and has considerable potential for application in clinical sample diagnostics. Real sample analysis also confirmed the successful detection of *Salmonella* Typhi in food and environmental samples, demonstrating its potential application in nucleic acid diagnostics of complex samples (Figure 6h; Figure S13, Supporting Information).

## 3 Conclusion

In this study, we proposed an enhanced qPCR technology by the compartmentalization and aggregation of biomolecular condensates in a crowded hydrogel, achieving rapid and highly sensitive nucleic acid detection. By adding two PEG monomers to the conventional PCR system, the hydrogel can be crosslinked within 2 min. The natural 3D network structure formed by the biomimetic hydrogel was similar to that of the intracellular environment, which enabled the concentration of PCR components in the nanoreactors, thereby enhancing the local concentration, collision, and enzyme reaction. By leveraging the aggregation of biomolecular condensates in the hydrogel, we proposed a mechanism of component enrichment and enhanced DNA polymerase activity to explain the significant improvement in PCR amplification efficiency. Compared with conventional aqueous solution PCR, primer‐template binding was enhanced by 1.37 times, and polymerase extension activity was increased by 4.87 times. Additionally, the qPCR amplification technology in crowded hydrogels demonstrated strong resistance to KCl inhibition, providing robust amplification in samples where inhibitory substances are difficult to remove. The duration time of crowded hydrogel‐enhanced qPCR requires only 26 min and achieves a LOD of 5.10 × 10^−1^ copies/µL, which was two orders of magnitude lower than that of conventional qPCR technology. We anticipate that this technology will significantly improve the sensitivity of nucleic acid detection and replication, thereby providing faster and more reliable solutions for nucleic acid diagnosis, gene expression quantification, and gene sequencing based on PCR amplification.

## 4 Experimental Section

### Materials and Reagents

Platinum SuperFi II DNA Polymerase, PureLink Microbiome DNA Purification Kit, and SYBR Green I were obtained from Thermo Fisher Scientific (Waltham, MA, USA). Hydrogel monomers, including 4 Arm‐PEG‐AC (average molecular weight [AMW], 10000) and SH‐PEG‐SH (AMW, 3400), were purchased from Laysan Bio (Arab, Alabama). The EvaEZ Fluorometric Polymerase Activity Assay Kit was obtained from Biotium Inc. (Hayward, CAA). Frame‐Seal Incubation Chambers were obtained from Bio‐Rad (Hercules, CA, USA). Brain heart infusion (BHI) and Luria‐Bertani (LB) broths were purchased from Hope Bio‐technology (Qingdao, China). The agarose was purchased from Tsingke Biotechnology (Beijing, China). The YeaRed nucleic acid gel stain (10000× in DMSO) was purchased from Yeasen Biotechnology (Shanghai, China). Tris(2‐carboxyethyl)phosphine was bought from Beyotime Biotechnology (Shanghai, China). The BSA‐FITC was bought from Solarbio Science and Technology Co. Ltd. (Beijing, China). Phosphate‐buffered saline was purchased from Macklin Biochemical Technology (Shanghai, China). EnGen *Lba*Cas12a was purchased from New England Biolabs (Ipswich, MA). *Lwa*Cas13a was purchased from GenScript Biotechnology (Nanjing, China). Sequences listed in Tables S1 and S2 (Supporting Information), SARS‐CoV‐2 RNA (containing the nucleoprotein [N] gene), KCl, and dNTPs were purchased from Sangon Biotechnology (Shanghai, China).

### Bacterial Culture and DNA Extraction

The bacteria used in this study were *Salmonella* Typhi CICC 10871, *Staphylococcus aureus* ATCC 25923, *Listeria monocytogenes* CICC 21633, and *Vibrio parahaemolyticus* ATCC 17802. All strains were activated in BHI broth overnight at 37 ± 1 °C with continuous shaking at 180 rpm. A single colony obtained from BHI agar plate was cultured in LB broth or BHI broth at 37 ± 1 °C for 12 h, with continuous shaking at 180 rpm. The bacteria were collected using centrifugation (7500 × g, 5 min) and resuspended in sterilized double‐distilled water (ddH_2_O). Bacterial genomic DNA was extracted according to the manufacturer's instructions.

### Characterization of the Hydrogel

The transmittance of the hydrogel was measured using a microplate reader (200 PRO; Tecan, Gr¨odig, Austria). Hydrogels with different concentrations were freeze‐dried using a lyophilizer (SCIENTZ‐10N/A, Ningbo SCIENTZ Biotechnology, China) under a vacuum (10.0 Pa) at −80 °C for 48 h. The functional groups of the hydrogels were characterized using an ATR FT‐IR spectrometer (NICOLET iS50FT‐IR, Thermo Fisher Scientific) with a scanning range of 32 times from wave number 4000 to 400 cm^−1^. To observe the nanoporous structure of the hydrogels, freeze‐dried hydrogels were sprayed with gold using an ion sputtering instrument (GVC‐2000, Gewei Instrument Co., Beijing, China) under a vacuum (1.0 Pa) at 15 mA for 60 s, and then observed using a field emission scanning electron microscope (ZEISS GeminiSEM 560, Carl Zeiss, Germany).

### PCR Amplification in Hydrogel

The PCR reaction (25 µL) in hydrogel was prepared with 1 × SuperFi II buffer, 0.2 µm forward primer, 0.2 µm reverse primer, 200 µm dNTPs, 1 × Platinum SuperFi II DNA Polymerase, samples containing target DNA, 10% hydrogel monomers (containing 6.4 mm of 4 arm‐PEG‐AC and 12.8 mm of SH‐PEG‐SH), and nuclease‐free water. For real‐time fluorescence qPCR, 1 × SYBR Green I was added. For in situ PCR amplification, additional 2 × SYBR Green I was added, and the mixture was placed in the frame‐sealed incubation chambers and sealed. The reaction mixture (25 µL) was placed in the dark at 25 °C for 2 min, during which the two PEG monomers formed a hydrogel via Michael addition reaction. Subsequently, qPCR was performed in QuantStudio 3 (Thermo Fisher Scientific), and in situ PCR was performed in GE4T (Bio‐Gener Technology, Hangzhou, China). The PCR parameters were as follows: 95 °C for 30 s and 35 cycles (95 °C for 5 s, 60 °C for 30 s). The conventional qPCR reaction performed in solution was the same as that for the above components, except that the hydrogel monomer was not added. After amplification, a fluorescence endpoint image of the frame‐sealed incubation chamber was obtained using an inverted fluorescence microscope (Leica DMi8, Leica Biosystems, Germany) under 488 nm excitation light.

### Photobleaching and Fluorescence Recovery

Photobleaching and fluorescence recovery experiments were performed to observe the diffusion of biomolecules in aqueous solutions and hydrogels. Commercial BSA‐FITC was used as a protein. dsDNA was obtained via PCR amplification and purified using a DNA gel extraction kit, and 1 × SYBR Green I dye was added to enable DNA fluorescence. The sample was placed under a 20 × magnification microscope and irradiated with a 488 nm laser for 10 min to create a circular fluorescence‐bleached area. Subsequently, the sample was observed under a 5 × magnification microscope and images were captured at different time points to track the recovery of fluorescence bleaching. The fluorescence recovery rate of the bleached area was quantified using the ImageJ software. The diffusion coefficients were calculated based on a previous study.^[^ ^48^ ^]^

### Commercial Digital PCR Detection

A commercial digital PCR kit was used to determine the precise concentrations of the target. A 25‐µL reaction mixture contained 1 × SuperFi II buffer, 0.2 µm forward primer, 0.2 µm reverse primer, 200 µm dNTPs, 1 × SYBR Green I, 1 × Platinum SuperFi II DNA Polymerase, samples containing target DNA, and nuclease‐free water. The mixture was loaded onto a microchamber chip using an injector (QuantStudio 3D Digital PCR, Thermo Fisher Scientific). After oil sealing, the prepared digital PCR chip was placed on a thermal plate instrument (GE4T plate amplification instrument, Bio‐Gener Technology) for PCR amplification. The thermocycling procedure was as follows: 95 °C for 30 s, and 35 cycles (95 °C for 5 s, 60 °C for 30 s). After amplification, the positive microchambers were counted using an inverted fluorescence microscope (Leica DMi8, Leica Biosystems) under 488 nm excitation light.

### ssDNA Extension

To explore the mechanism of PCR enhancement, an ssDNA extension experiment was designed. The ssDNA extension mixture (25 µL) contained 1 × SuperFi II buffer, 1 µm reverse primer, 200 µm dNTPs, 0.25 × Platinum SuperFi II DNA Polymerase, 0.5 µm ssDNA template, 10% hydrogel monomers (containing 6.4 mm of 4 arm‐PEG‐AC and 12.8 mm of SH‐PEG‐SH), and nuclease‐free water. After allowing to stand for 2 min to form the hydrogel, the mixture was placed in a thermal cycler (Veriti 96‐well rapid thermal cycler; Thermo Fisher Scientific, Waltham, MA, USA) for the ssDNA extension reaction. The extension program was as follows: 95 °C for 5 min and 60 °C for different times from 0 to 60 min. The same mixture without hydrogel monomers, simultaneously underwent the ssDNA extension reaction in an aqueous solution. The extension products were quantified using agarose gel electrophoresis. In addition, the binding of primer‐template was quantified using the same method, but without the 60 °C extension step after the 95 °C denaturation.

### Agarose Gel Electrophoresis

PCR amplification products, ssDNA extension products, and prime‐template binding were detected using agarose gel electrophoresis. Samples were electrophoresed on a 3% (w/v) agarose gel at 140 V for 36 min (ELITE 300, WEALTEC, NV, USA). Images were acquired at a fixed exposure time using a gel imaging system (SH‐510; SHST, Hangzhou, China). Band intensity was quantified using ImageJ software. The obtained images were first converted to 8‐bit format and the background was subtracted using the “Subtract Background” function (rolling ball radius set to 50 pixels). Each band was selected using a rectangular selection tool, and the peak area of the resulting histogram was measured to represent the band intensity.

### Real Sample Detection

Clinical samples were collected and analyzed in accordance with the ethical guidelines and regulations, were approved by the Ethics Committee of the Children's Hospital of Zhejiang University School of Medicine (2023‐IRB‐0243‐P‐01), and written informed consent was obtained from all participants. Blood, urine, and saliva samples from healthy individuals were previously analyzed by the Clinical Microbiology Laboratory at the Children's Hospital of Zhejiang University School of Medicine. Milk, fruits and vegetables were purchased from local markets, and lake water samples were collected from Zhejiang University. All samples were analyzed using standard qPCR and hydrogel qPCR assays.

### Statistical Analysis

All the assays were performed in triplicate. All data were analyzed using Excel and results were represented as mean ± standard deviation, unless otherwise noted. Statistical analysis was performed using the two‐tailed Student's *t*‐test among two groups and one‐way analysis of variance was applied to multiple treatment comparison. *p* < 0.05 was considered statistically significant. All graphs were drawn using OriginPro 2024 software.

## Conflict of Interest

The authors declare no conflict of interest.

## Supporting information



Supporting Information

## Data Availability

The data that support the findings of this study are available from the corresponding author upon reasonable request.
