# Supplementary material for: Compartmentalization and Aggregation of Biomolecular Condensates in Crowded Hydrogels for Enhanced Nucleic Acid Diagnosis
Source: Adv Sci (Weinh). 2025 Nov 5;13(3):e13938. doi: 10.1002/advs.202513938 (PMC12806502; doi:10.1002/advs.202513938)
Supplement: Supplementary file 1 — Supporting Information [file ADVS-13-e13938-s001.pdf]

Supplementary Information

Compartmentalization and Aggregation of Biomolecular Condensates in Crowded Hydrogels for Enhanced Nucleic Acid Diagnosis

Fangbin Xiao [a,b], Tao Yang [a], Mei Fang [a], Xingyu Lin [a,b*]

[a] College of Biosystems Engineering and Food Science, Zhejiang University, Hangzhou, 310058, China

[b] Binjiang Institute of Zhejiang University, Hangzhou 310053, China

Correspondence to Dr. Xingyu Lin, Email address: xingyu@zju.edu.cn

Table S1. Sequences used for PCR amplification.

Trget	Gene	Gene Bank	Sequence (5’-3’)	Amplicon length
Salmonella Typhi	*sii*A	CP117294.1	F-ACGACTGGGATATGAACGGGGAA<br>R-TCGTTGTACTTGATGCTGCGGAG	109 bp
Salmonella Typhi	STY3098		F-TTTGGCGGCGCAGGCGATTC<br>R-GCCTCCGCCTCATCAATCCG	423 bp
Salmonella Typhi	*inv*A	CP149380.1	F-AAACCTAAAACCAGCAAAGG<br>R-TGTACCGTGGCATGTCTGAG	605 bp
Listeria monocytogenes	*inl*A	NC_003210.1	F-ACGAGTAACGGGACAAATGC<br>R-CCCGACAGTGGTGCTAGATT	800 bp
ssDNA template	*sii*A	AJ576316.1	ATATCCTGATGTAGTTATTGACATGA<br>GTGTTAACTCCGCAGCATCAAGTAC<br>AACGAG	-

Table S2. Sequences used for CRISPR/Cas12a and CRISPR/Cas13a reactions.

Name	Target	Sequence (5'-3')
Forward primer	16S rDNA	TCCTACGGGAGGCAGCAGT
Reverse primer	16S rDNA	GGACTACCAGGGTATCTAATCCTGTT
Vp-crRNA	16S rDNA	UAAUUUCUACUCUUGUAGAUAUAGCGUAUUCGUUUGACGUUAA
N-crRNA	N gene	GACCACCCCAAAAAUGAAGGGGACUAAAACUUUGCGGCCAAUGUUUGUAA
ssDNA-reporter		FAM-CCCCCC-BHQ1
ssRNA-reporter		FAM-UUUUU-BHQ1

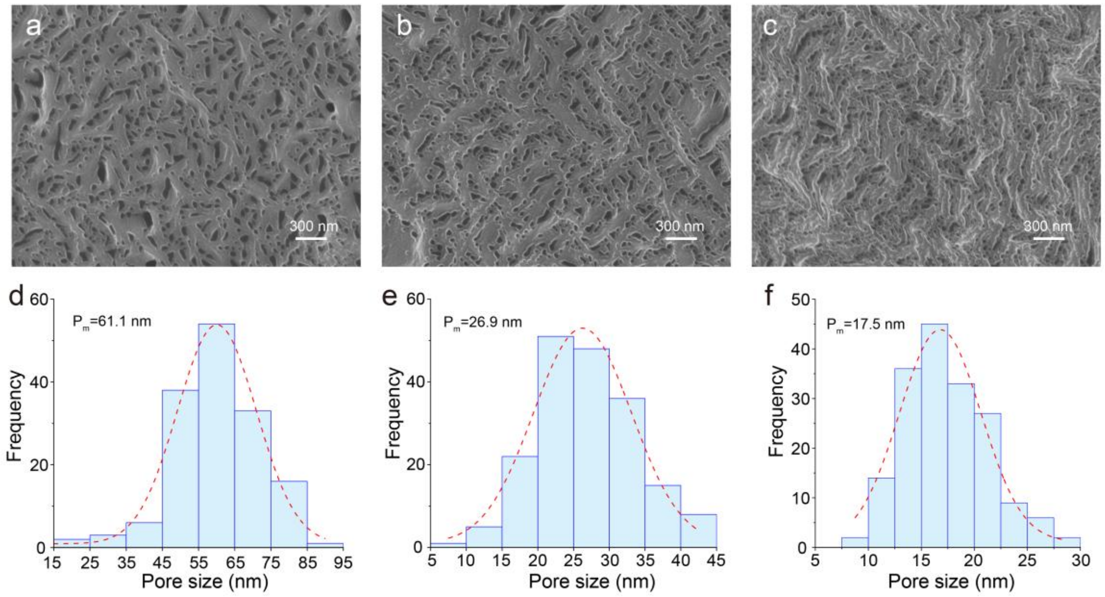

Figure S1. SEM images of nanoporous hydrogel with concentrations of 5% (a), 10% (b), 15% (c). Pore size distribution histogram of hydrogels with concentrations of 5% (d), 10% (e), 15% (f).

Primer recruitment by condensates in hydrogel

To confirm the presence of recruitment behavior by condensates, FAM (5-Carboxyfluorescein, a green fluorescent reagent used for labeling nucleotides) labeled primer was used. If FAM-labeled primers are evenly distribtuted in the hydrogel, a uniform green background will be obtained. However, as shown in **Figure S2**, the FAM-labeled primers tended to aggregate after the amplification and formed distinct fluorescent spots, demonstrating that condensates within the hydrogel have the function of recruiting and enriching DNA. By quantifying the fluorescence intensities of the background and condensates, we found that the FAM-labeled primers were enriched by 3.08-fold.

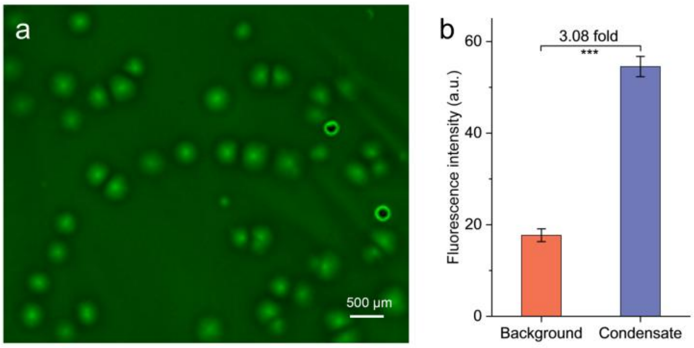

Figure S2. Primer recruitment by condensates in hydrogel. (a) Fluorescence images of primer recruitment by condensates in hydrogel. (b) Fluorescence intensity quantification of condensates and background (Bars represent the mean ± SD, n = 3), and significant difference was determined by a two-tailed student's t-test (*$P < 0.05$; **$P < 0.01$; ***$P < 0.001$; ****$P < 0.0001$; ns: $P \geq 0.05$).

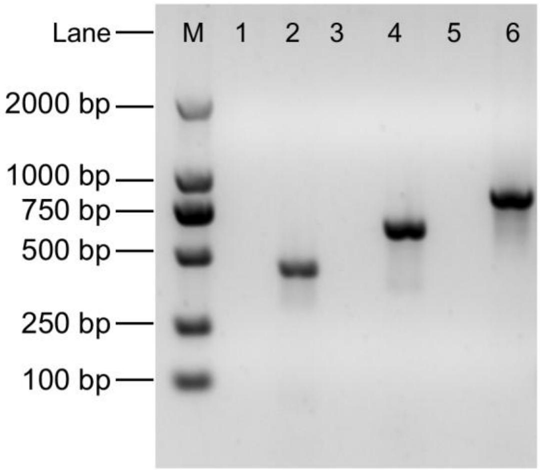

Figure S3. Agarose gel electrophoresis of DNA condensates with different lengths. Lane M, 2,000 bp DNA Marker. Lanes 1 and 2 are extracted from the background and fluorescent dots in hydrogel with a length of 423 bp, lanes 3 and 4 are extracted from the background and fluorescent dots in hydrogel with a length of 605 bp, lanes 5 and 6 are extracted from the background and fluorescent dots in hydrogel with a length of 800 bp.

Combination of primer and template

The secondary structures and free energy of ssDNA template and reverse primer (Rv-primers) at specific temperatures were predicted using NUPACK software (https://nupack.org/) **(Figure S4)**. The calculated free energies were -0.65 kcal/mol for the Rv-primer and -8.15 kcal/mol for ssDNA, suggesting that ssDNA is more likely to form secondary structures at room temperature. After the mixture was denatured at 95°C and annealed to 60°C, the free energy of the Rv-primer-ssDNA binding was -22.75 kcal/mol, indicating a strong binding force between the Rv-primers and the ssDNA template.

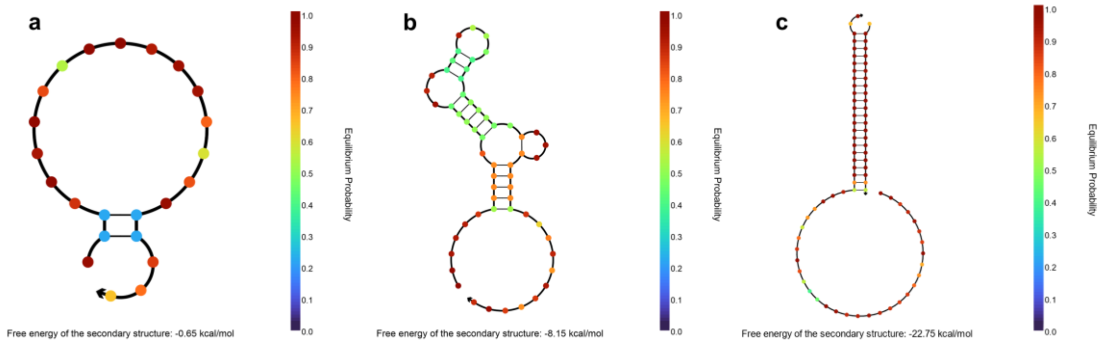

Figure S4. Minimum free energy (MFE) proxy structure of Rv-primer (a) at 25°C, ssDNA template (b) at 25°C, and combination of Rv-primer with ssDNA template (c) after desaturating and annealing at 60°C, predicted by NUPACK software (https://nupack.org/).

DNA polymerase activity assay

A commercial kit was used to measure the DNA polymerase activity in hydrogel and aqueous solution. The fluorescence intensity represents the amount of double-stranded DNA product synthesized. As shown in **Figure S5**, in the initial stage of the reaction, the synthesis of deoxynucleotides increased at a linear rate and was completed within 60 minutes. By fitting the slope of the initial stage of the real-time fluorescence curve, we found that the nucleotide synthesis rate in 10% PEG was 2.15-fold that in aqueous solution, indicating that the hydrogel could significantly improve the extension activity of DNA polymerase. In addition, we observed a similar DNA polymerase activity enhancement in a system containing 50 mM of KCl inhibitor.

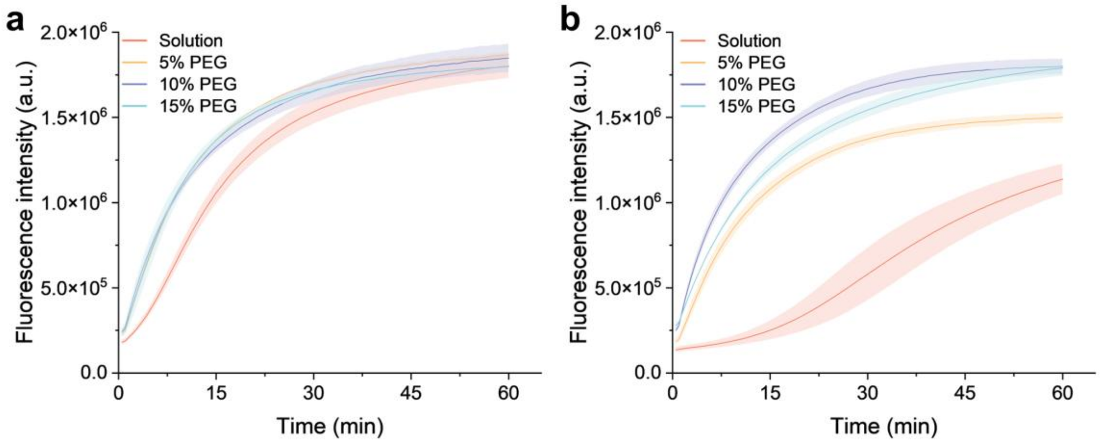

Figure S5. Measurement of DNA polymerase activity in solution without (a) or with (b) 50 mM KCl inhibitor using a commercial kit (Color-filled bars represent the mean ± SD, *n* = 3).

Cas12a and Cas13a reaction in hydrogel

Molecular diagnostic methods based on CRISPR/Cas systems have been widely developed due to their excellent specificity and signal amplification effect. We investigated the trans-cleavage kinetics based on Cas12a and Cas13a in spatially confined hydrogels. As a proof-of-concept, we initially amplified the 16S rDNA of bacteria using universal primers and designed specific CRISPR RNA (crRNA) targeting the V3-V4 region of *Vibrio parahaemolyticus* (**Table S2**), followed by the detection of amplicons based on the classical CRISPR/Cas12a system. The CRISPR/Cas12a detection system in 25 μL of mixture in hydrogel including 1 × reaction buffer (containing 10 mM of Tris-HCl at pH 8.7, 10 mM of NaCl, 15 mM $MgCl_2$, and 1 mM DTT), 200 nM of *Vp*-crRNA, 100 nM of Cas12a, 400 nM of ssDNA reporter, 2 μL of 16S rDNA amplicons, 4% hydrogel monomers (containing 0.64 mg 4 arm-PEG-AC and 0.44 mg SH-PEG-SH), and nuclease-free water. The reactions were monitored in QuantStudio 3 (Thermo Scientific, Waltham, MA, USA) at 37 °C with fluorescence measurements (FAM channel) taken every 1 minute, and the total time is 60 min.

The RNA detection based on CRISPR/Cas13a system was also performed in hydrogel, while N gene of SARS-CoV-2 was chosen as the target. Briefly, reaction (25 μL) was prepared as 1 × reaction buffer (containing 10 mM of Tris-HCl at pH 8.7, 50 mM of KCl, and 1.5 mM of $MgCl_2$), 50 nM of N-crRNA, 50 nM of Cas12a, 800 nM of ssRNA reporter, 2 μL of N gene RNA (1260 nt), 5% hydrogel monomers (containing 0.8 mg 4 arm-PEG-AC and 0.55 mg SH-PEG-SH), and DEPC water. The reactions were monitored in QuantStudio 3 (Thermo Scientific, Waltham, MA, USA) at 37 °C with fluorescence measurements (FAM channel) taken every 1 minute, and the total time is 60 min.

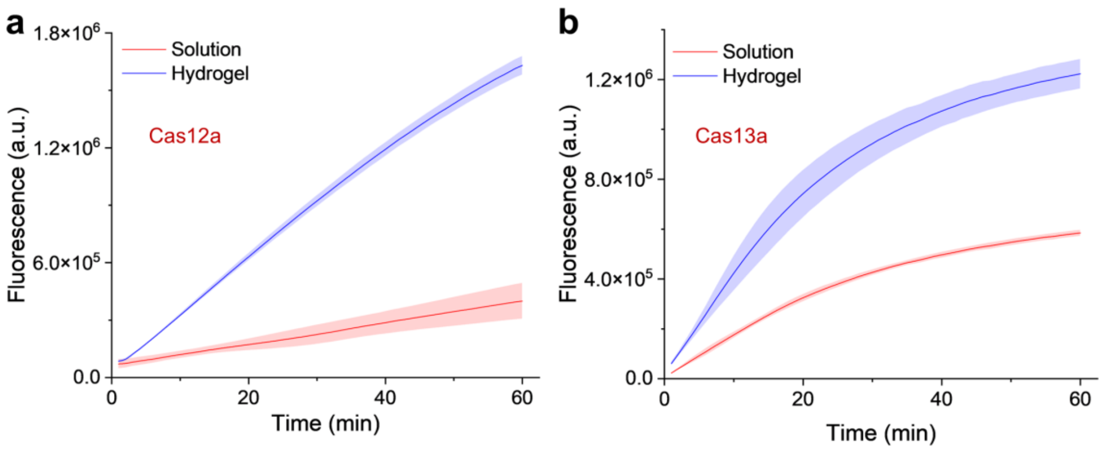

Figure S6. Cas12a (a) and Cas13a (b) reactions in solution and hydrogel systems (Color-filled bars represent the mean ± SD, $n = 3$).

PCR amplification in different systems

In order to determine whether the PCR enhancement phenomenon is caused by the compartmentalization and crowding of condensates in the hydrogel, we performed PCR amplification in three systems including a hydrogel system, a system containing two PEG monomers but not gelling, and an aqueous solution system. As shown in **Figure S7**, obvious DNA condensate spots were formed in the hydrogel system, and the Ct value was significantly earlier than that of the non-gelling system and the aqueous solution system. These results indicated that PEG and gelation were necessary for PCR enhancement.

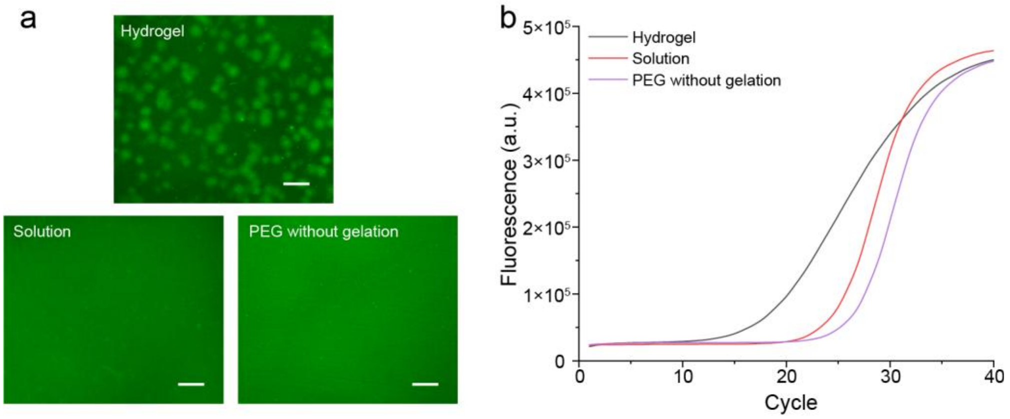

Figure S7. PCR amplification in different systems. (a) Fluorescence images of PCR amplification in different systems. Scale bar, 500μm. (b) Real-time fluorescence quantification curves of PCR amplification in different systems.

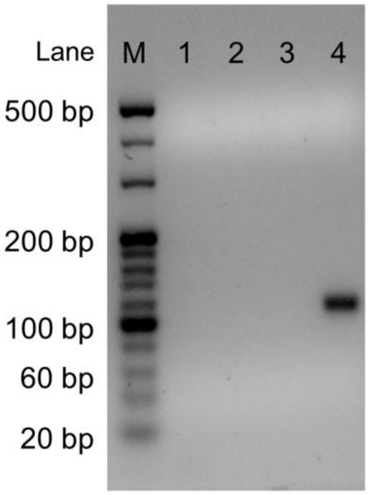

Figure S8. Agarose gel electrophoresis image of fast real-time fluorescence quantitative amplification products. Lanes M, 20 bp DNA ladder. Lanes 1 to 4 represented the amplified products in solution (negative), solution (positive), hydrogel (negative), and hydrogel (positive), respectively.

Repeatability, stability, and compatibility of hydrogel-enhanced PCR

To assess the repeatability of hydrogel-enhanced PCR, we performed eight independent experiments (each with three replicates). As shown in **Figure S9**, the qPCR amplification curves in both solution and hydrogel systems were highly consistent. The mean Ct values of solution and hydrogel systems were 22.16 and 18.57, respectively, showing a statistically significant difference. These results demonstrated that hydrogel-enhanced PCR exhibited high repeatability comparable to that of conventional solution PCR.

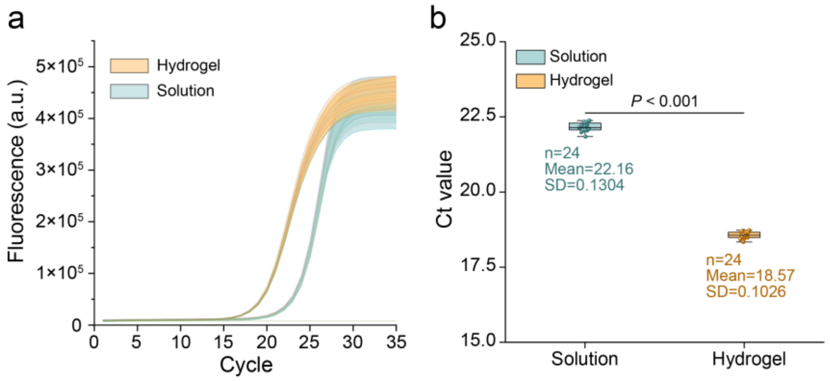

Figure S9. Repeatability test. (a) Real-time fluorescence quantitative curves of aqueous solution and hydrogel PCR (n = 24). (b) Corresponding Ct values of aqueous solution and hydrogel PCR (Bars represent the mean ± SD, n = 24), and significant difference was determined by a two-tailed student's t-test ($P < 0.05$ was considered statistically significant). Mean represents the average Ct value, SD denotes standard deviation.

Hydrogel stability is essential for ensuring consistent detection performance. As shown in **Figure S10a**, the hydrogel remained transparent and retained its original morphology after being subjected to different numbers of PCR cycles. Even when the PCR tube was inverted, the hydrogel at the bottom did not detach or deform. Furthermore, to evaluate long-term stability, pre-formed hydrogel PCR mixtures were stored at 25°C for varying durations before qPCR amplification. Results in **Figure S10b** indicated that the hydrogel qPCR maintained its initial detection performance after storage for up to 72 h under near-ambient conditions, with a coefficient of variation (CV) of Ct values as low as 0.01438. These findings confirmed that the hydrogel PCR system possessed excellent stability and could be stored for extended periods without

compromising detection performance.

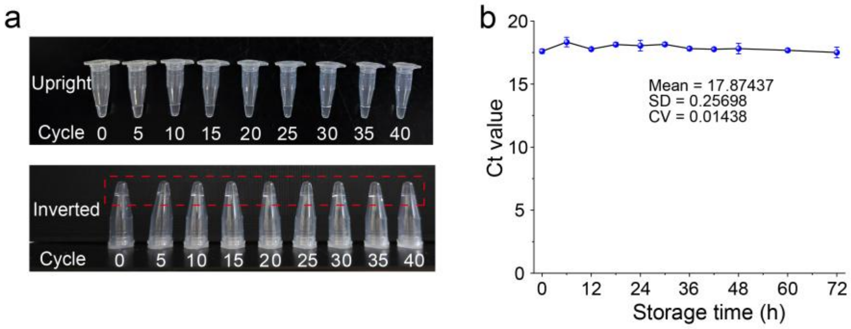

Figure S10. Stability assessment of the hydrogel. (a) Images of hydrogels subjected to different numbers of PCR cycles. (b) Detection performance of hydrogel PCR after different storage periods (Bars represent the mean ± SD, n = 3). Mean indicates the average Ct value, SD represents standard deviation, CV corresponds to the coefficient of variation.

Finally, we examined the hardware compatibility of hydrogel PCR. As shown in **Figures S11a–c**, the same qPCR assay was carried out using StepOne (Applied Biosystems, Waltham, MA, USA), QuantStudio 3 (Thermo Scientific, Waltham, MA, USA), and QuantStudio 5 (Thermo Scientific, Waltham, MA, USA) instruments. The absolute fluorescence values detected by the three systems differed, likely due to variations in the sensitivity of their respective fluorescence detection systems. However, this did not affect the Ct values, which typically served as the key parameter for evaluating qPCR performance (**Figure S11d**). In addition, our hydrogel PCR was compatible with common PCR hardware, including standard plates, eight-tube strips, and *in situ* PCR chambers (**Figure S11e**). These results demonstrated that hydrogel PCR offered broad hardware compatibility and could be readily applied to most commercial PCR platforms without loss of detection performance.

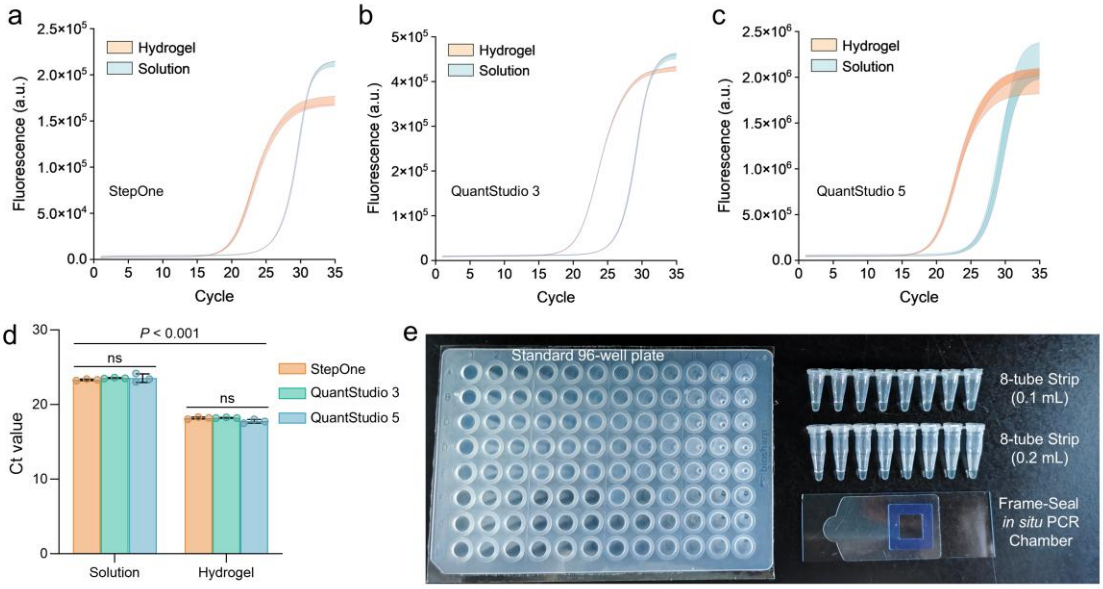

Figure S11. Compatibility of hydrogel-enhanced PCR. Real-time fluorescence curves of solution and hydrogel qPCR performed on StepOne (a), QuantStudio 3 (b), and QuantStudio 5 (c) systems ($n = 3$). (d) Corresponding Ct values of solution and hydrogel qPCR in different instruments (Bars represent the mean ± SD, $n = 3$), and significant difference was determined by one-way ANOVA with multiplicity adjusted P value (*$P < 0.05$; **$P < 0.01$; ***$P < 0.001$; ****$P < 0.0001$; ns: $P \geq 0.05$). (e) Photograph of hydrogel PCR compatibility with various common PCR hardware.

Clinical sample testing

To further validate the stability of hydrogel-enhanced PCR in complex matrices, we evaluated its performance in blood, urine, and saliva samples. **Figure S12** shows the real-time fluorescence amplification curves of both conventional solution PCR and hydrogel PCR, with control groups containing no clinical samples. As demonstrated, the detection performance of hydrogel-enhanced PCR remained consistent across tests involving various concentrations of blood, urine, and saliva, with no significant delay in Ct values. In contrast, conventional PCR amplification in aqueous solution exhibited varying degrees of inhibition in the presence of these clinical samples. It should be noted that a noticeable decrease in fluorescence signal was observed during real-time PCR detection in blood samples (**Figure S12a**), which can be attributed to the intrinsic color of blood interfering with fluorescence measurement. However, this effect did not notably alter the Ct values in the hydrogel-based system (**Figure 6g**).

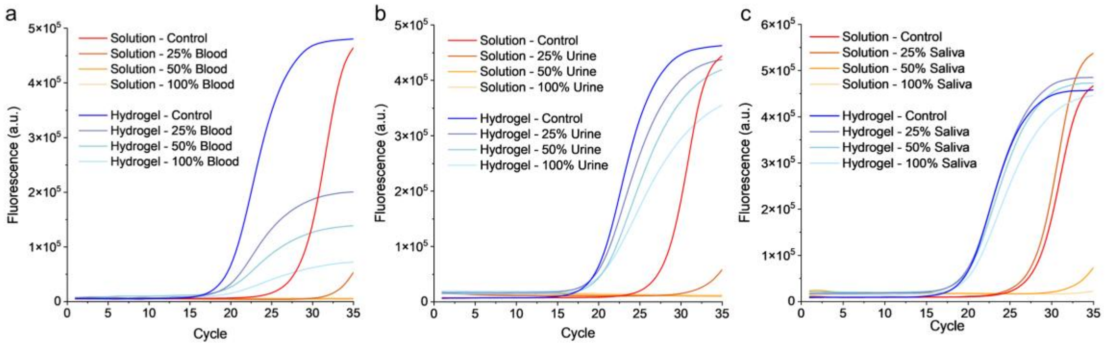

Figure S12. Effects of different concentrations of blood (a), urine (b), and saliva (c) clinical samples on qPCR in solution and hydrogel.

Amplicon sequencing alignment of real samples

To confirm that the nucleic acid detected in the Lake Water 1 and Lake Water 3 samples corresponded to *Salmonella*, we performed Sanger sequencing of the amplified PCR products. Sequencing alignment results are shown in **Figure S13**. The sequences obtained from both Lake Water 1 and Lake Water 3 matched perfectly with the *sii*A gene sequence of *Salmonella* Typhi, confirming that the detected targets in these two samples were indeed *Salmonella* Typhi.

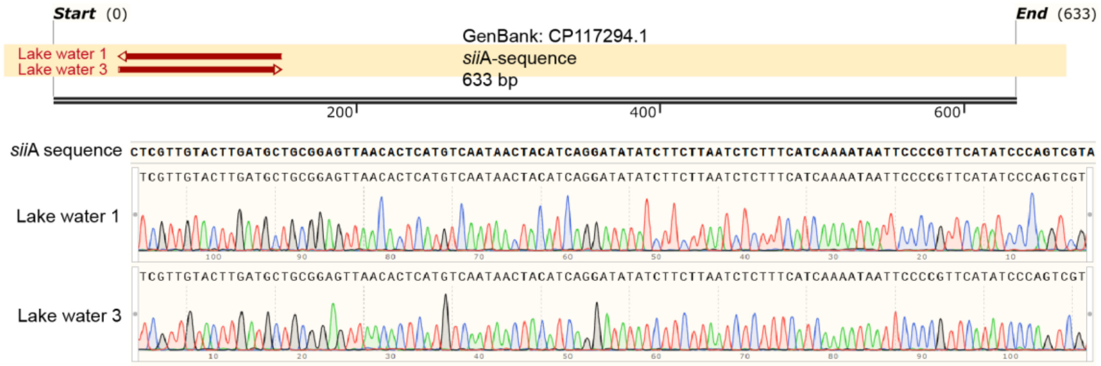

Figure S13. Amplicon sequencing alignment of real samples.
